# Hyperglycemia Reduces Efficiency of Brain Networks in Subjects with Type 2 Diabetes

**DOI:** 10.1371/journal.pone.0157268

**Published:** 2016-06-23

**Authors:** Dae-Jin Kim, Ji Hee Yu, Mi-Seon Shin, Yong-Wook Shin, Min-Seon Kim

**Affiliations:** 1 Department of Psychological and Brain Sciences, Indiana University, Bloomington, Indiana, United States of America; 2 Division of Endocrinology and Metabolism, Department of Internal Medicine, Korea University College of Medicine, Ansan, Korea; 3 Division of Endocrinology and Metabolism, Department of Internal Medicine, KEPCO Medical Center, Seoul, Korea; 4 Department of Psychiatry, Ulsan University School of Medicine, ASAN Medical Center, Seoul, Korea; 5 Division of Endocrinology and Metabolism, Ulsan University College of Medicine, Seoul, Korea; 6 Diabetes Center, ASAN Medical Center, Seoul, Korea; Universidad Rey Juan Carlos, SPAIN

## Abstract

Previous research has shown that the brain is an important target of diabetic complications. Since brain regions are interconnected to form a large-scale neural network, we investigated whether severe hyperglycemia affects the topology of the brain network in people with type 2 diabetes. Twenty middle-aged (average age: 54 years) individuals with poorly controlled diabetes (HbA1c: 8.9−14.6%, 74−136 mmol/mol) and 20 age-, sex-, and education-matched healthy volunteers were recruited. Graph theoretic network analysis was performed with axonal fiber tractography and tract-based spatial statistics (TBSS) using diffusion tensor imaging. Associations between the blood glucose level and white matter network characteristics were investigated. Individuals with diabetes had lower white matter network efficiency (*P*<0.001) and longer white matter path length (*P*<0.05) compared to healthy individuals. Higher HbA1c was associated with lower network efficiency (*r* = −0.53, *P* = 0.001) and longer network path length (*r* = 0.40, *P*<0.05). A disruption in local microstructural integrity was found in the multiple white matter regions and associated with higher HbA1c and fasting plasma glucose levels (corrected *P*<0.05). Poorer glycemic control is associated with lower efficiency and longer connection paths of the global brain network in individuals with diabetes. Chronic hyperglycemia in people with diabetes may disrupt the brain’s topological integration, and lead to mental slowing and cognitive impairment.

## Introduction

Diabetes is a chronic, metabolic disease characterized by hyperglycemia which leads over time to serious damage to the blood vessels in eyes, kidneys, nerves, and heart. Diabetes is classified into type 1 (IDDM, insulin dependent diabetes mellitus), type 2 (NIDDM, non-insulin dependent diabetes mellitus), and gestational (temporal insulin resistance during pregnancy). Type 2 diabetes mellitus (T2DM) is the most common form of diabetes in adults and its prevalence has been sharply increasing during the past three decades. T2DM are at greater risk of cognitive dysfunction, vascular dementia, and Alzheimer’s disease [1]. They also display concurrent structural changes in the brain such as cortical atrophy, deep white matter hyperintensities, and lacunar infarctions. Diabetes-related cognitive impairment might be attributable to these structural changes, because white matter (WM), which consists of axonal fibers, has a pivotal role in transferring information between distributed cortical regions, and the functional efficiency of the brain highly depends on the microstructural integrity of WM connecting brain regions. Recent magnetic resonance imaging (MRI) technology has revealed such microstructural changes in the WM of people with diabetes. Specifically, using diffusion tensor MRI (DT-MRI), significant alterations of WM integrity were demonstrated, in particular, in the corpus callosum, the internal and external capsules, and posterior cerebral regions in people with T2DM and metabolic syndrome [2–6]. Disrupted WM integrity, furthermore, was shown to be related to the reduced cognitive ability (*e*.*g*., executive function) in these subjects [6, 7].

The cortical regions are functionally distributed across the brain and highly interconnected by axonal fibers, generating a complex brain network. Connections among those regions could be defined by the anatomical links (= structural connectivity), statistical dependencies using temporal correlations (= functional connectivity), or causal interactions (= effective connectivity), giving different modes of brain network. The structural brain network has been primarily formulated using graph theory, a mathematical description in which the brain is represented as a set of nodes (*i*.*e*., anatomically distinct brain regions) and edges (*i*.*e*., axonal WM tracts connecting nodes), the so-called structural *connectome* [8, 9]. A node may represent a single neuron, a set of neurons, or an anatomical region of the brain. An edge refers to the connection between two nodes, which can be either binary or weighted, and either directed or undirected. The idea of the connectome implies that the human brain is highly-segregated (*i*.*e*., brain regions responsible for similar functions are anatomically clustered.) and highly-integrated (*i*.*e*., remote brain regions interact efficiently.) [10]. The topological properties of a network can be quantitatively described by a number of mathematical measures that can focus on network segregation (*e*.*g*., clustering coefficient and modularity), network integration (*e*.*g*., characteristic path length and efficiency), and the balance between the two (*e*.*g*., small-worldness).

So far, one study has used graph theoretical analysis to examine alterations in the structural brain network of diabetic patients, in which the cerebral WM network was disrupted in terms of whole-brain network segregation and integration in T2DM [11]. However, the subjects in the study were elderly (mean age, 71 ± 4 years) and their HbA1c values were low enough (6.7 ± 0.7%) to establish an impact of hyperglycemia on the brain’s structural network. In the present study, we investigated structural brain network in middle-aged people with poorly controlled T2DM. We also investigated correlations of blood glucose level with measures of network topology and local microstructural property of WM.

## Materials and Methods

### Participants

Twenty Korean adults between 30 and 70 years of age with poorly controlled T2DM (HbA1c >8%), and age-, sex-, and education-matched 20 healthy volunteers (all right-handed) were recruited for the study (Table 1). Participants with T2DM were recruited through physician referrals from the ASAN Diabetes Center, Seoul, Korea. Control participants were recruited using flyers and advertisements. One week before brain imaging, a screening interview was conducted with each participant to obtain information from his/her medical records. For all participants, measurements of blood pressure, height, and body weight were recorded during the screening sessions. Exclusion criteria included history of serious head injury (with loss of consciousness >5 min), transient ischemic attack or stroke, other neurological illness, psychiatric disorder, heart disease, learning disability, severe hypoglycemia, alcohol or substance dependence in the previous three months, and intoxication via urine screen at the time of testing. After providing a complete description of the study, written informed consent was obtained from all participants. The research protocol was approved by the Institutional Review Board of ASAN Medical Center.

**Table 1:** Demographic characteristics of the subjects.

	Controls	T2DM
*n*	20	20
Age (years)	54.3 ± 2.4	54.6 ± 2.3
Number of male (%)	9 (45)	9 (45)
Height (cm)	165.2 ± 6.9	163.8 ± 8.5
Weight (kg)	64.5 ± 6.8	66.3 ± 10.1
BMI (kg/m^2^)	23.6 ± 0.4	24.7 ± 0.6
Education (years)	10.0 ± 3.6	11.9 ± 2.3
Systolic blood pressure (mmHg)	118 ± 15	126 ± 14
Diastolic blood pressure (mmHg)	72 ± 14	72 ± 9
Former or current smoker (*n*, *%*)	6 (30)	11 (55)*
Total cholesterol (mg/dl)	185 ± 40	167 ± 52
FPG (mmol/L) [normal range: 4–5.5 mmol/L]	5.19 ± 0.13	10.0± 1.04**
HbA1c (%) [normal range: <6%]	5.9 ± 0.1	10.7 ± 0.3**
HbA1c (mmol/mol)	40.9 ± 0.7	93 ± 2.6**
Duration of diabetes (years)		12.1 ± 6.5
Diabetic retinopathy (*n*, %)		9 (45)
Diabetic nephropathy (*n*, %)		4 (20)
Diabetic peripheral neuropathy (*n*, %)		7 (35)

Data are represented as mean ± SD *or n* (%).

* *P* <0.05

**P <0.005 *vs*. control.

*Abbreviations*: BMI, body mass index; FPG, fasting plasma glucose; HbA1c, glycated hemoglobin A1c.

Demographic characteristics of the participants are summarized in Table 1. Participants in both groups were moderately well-educated, without symptoms of depression, of average intelligence, and middle-aged. No group differences were found in age, sex, body mass index, blood pressure, or plasma cholesterol levels. The percentage of former or current smokers was higher in the group with diabetes than in the control group. Mean HbA1c in the group with diabetes was 10.7% (range 8.9−14.6%, 74−136 mmol/mol) and mean duration of diabetes was 12 years (range 5−28 years). Nine of the 20 patients (45%) had diabetic retinopathy, 4 (20%) had diabetic nephropathy, and 7 (35%) had diabetic peripheral nephropathy. Diagnosis of diabetic retinopathy was done based on retinal examination by trained ophthalmologists. Diabetic nephropathy was defined by the presence of albuminuria [12] and diabetic neuropathy by symptoms and signs of distal symmetrical peripheral neuropathy based on the examination of a neurologist and abnormal nerve conduction findings in ≥ 2 anatomically distinct nerves among the sural, peroneal and median nerves [13].

### MRI acquisition

Whole brain MRI scans were collected with a Siemens Magnetom TrioTim 3T scanner between 9 and 12 AM following overnight fasting. Prior to image acquisition, fasting plasma glucose (FPG), HbA1c, and cholesterol levels were measured by enzymatic methods using an auto-analyzer (Hitachi E170, Hitachi, Ltd., Tokyo, Japan). FPG was also measured using a glucometer to ensure it was over 80 mg/dl during the scan. Diffusion tensor images (DTI) with a spin-echo echo-planar imaging (SE-EPI) sequence were acquired twice with 30-directional diffusion weighted images including one non-diffusion weighted image and averaged to increase the signal-to-noise ratio with: *b* = 1,000 s/mm^2^, repetition time (TR) = 4,700 ms, echo time (TE) = 87 ms, field of view = 240×240 mm, image matrix = 128×128, 1.88×1.88×4 mm^3^ voxels with 37 transverse slices during about 4 min 52 sec. The anisotropic voxel was resampled to have a size of 1.88×1.88×2 mm^3^. In addition, anatomical T1-weighted MRI scans were acquired using a T1 turbo flash echo sequence with: 256×256 image matrix with 192 sagittal slices, 1 mm isocubic voxels, TE 2.52 ms, TR 1,900 ms and flip angle 9°. The duration of this imaging sequence was 6 min 15 sec. None of the participants showed visible abnormal findings in their structural MRI scans including T1-hyperintense legion.

### MRI preprocessing

**Fig 1:**
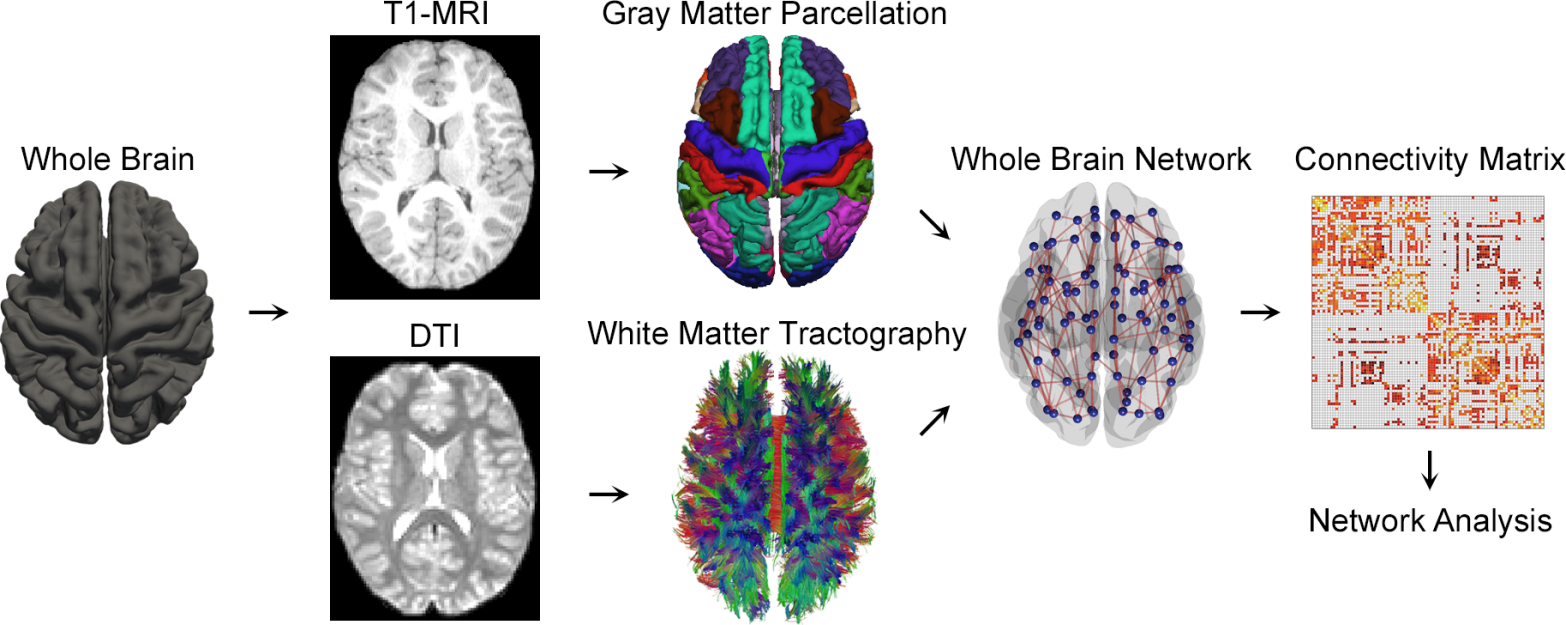
Diagram of the brain network analysis. T1-weighted anatomical MRI scans of each individual were used to parcellate the cortex into 144 brain regions, forming the nodes of a brain network. Whole brain fiber tractography was applied to the DTI scans to reconstruct white matter pathways connecting pairs of brain regions, and the network edge was defined by the fiber density between the regions. The structural network was constructed in the form of a 144 × 144 weighted symmetric connectivity matrix. The topological organization of the resulting brain networks, including the correlations with blood glucose level (HbA1c and FPG), was investigated for 20 persons with type 2 diabetes and their age- and sex-matched controls.

Preprocessing of MR images was performed using FSL toolbox (http://fsl.fmrib.ox.ac.uk/fsl/fslwiki) [14–16] and Freesurfer (http://freesurfer.net) [17, 18] as in Fig 1. The steps included: (1) visual inspection for MRI artifacts, (2) removal of non-brain regions, (3) correction for eddy-current and head motion, (4) estimation of diffusion tensor, and (5) coregistration to the T1-weighted anatomical image. Voxel-wise diffusion parameters such as fractional anisotropy (FA, a measure of the directional coherence for the fiber tracts), mean diffusivity (MD, the average magnitude of molecular displacement by diffusion), axial diffusivity (AD, a length of the longest axis of diffusion tensor), and radial diffusivity (RD, the averaged length of two remaining axis of diffusion tensor) were computed. T1-weighted MRI was segmented into gray/whiter matter and cerebrospinal fluid. The segmented gray matter was partitioned into 144 anatomic regions of interest (ROIs) to represent nodes of the individual brain networks using Destrieux atlas of Freesurfer [19].

### Structural connectivity and the brain network

Whole brain deterministic fiber tractography was performed in the native DTI space with Diffusion Toolkit (http://trackvis.org) [20]. To start fiber tracking, twenty seeds randomly distributed within each voxel were used to generate a sufficient number of fiber tracts. All fiber tracts were computed for voxels with FA >0.2 and a smooth turn of <30°. The brain was modeled as a set of nodes and edges using graph theory [8], where nodes represent the parcels of gray matter, and the edge represents WM tracts interconnecting two nodes. Unbiased methods to find fiber tracks between brain regions have long been debated [21]. Here the presence of an edge connecting two brain regions was defined if there were at least three fiber tracts between them to reduce false-positive connections [22, 23]. Structural connectivity as a network edge was defined as a weight representing the number of tractography streamlines connecting two node regions, normalized by the number of whole fiber tracts in the brain–*i*.*e*., fiber density. This resulted in a 144×144 weighted symmetric connectivity matrix.

### Global characteristics of the structural brain network

Topological characteristics of the structural brain network were analyzed using Brain Connectivity Toolbox (http://sites.google.com/site/bctnet). In this study, the network characteristics of each participant were investigated with respect to network segregation–clustering coefficient (γ) and modularity (Q), integration–path length (λ) and efficiency (E), and their optimal balance (small-worldness, σ) [8]. Mathematical details of the network implications can be found in previous studies [22, 24]. The network segregation and integration could also be determined by other descriptive measures such as motif and information capability [25].

#### Network segregation

In biological networks including the human brain, functional interactions within the network are assumed to increase as nodes get topologically closer [8]. Network segregation represents the extent to which closely and densely coupled neighbors form local clusters or modules in the network. In this study, the clustering coefficient [26] and modularity [27] were chosen as measures of network segregation [24]. First, the clustering coefficient of each node was computed as the likelihood that the neighbors of a node are interconnected to each other, then the clustering coefficients were averaged to yield a scalar measure for global network clustering. Following that, the computed clustering coefficient was normalized by the average of the clustering coefficients in a population of 1,000 randomized null networks preserving the local node structure but with randomized global topology [24]. The normalized clustering coefficient (γ) quantifies the extent to which the network is organized into densely segregated nodes with respect to the random networks. Second, a module in the network can be defined as a subdivision that has more connections within the module than outside it. Modularity (Q) quantifies the degree to which the network can be optimally partitioned into distinct subcommunities [24].

#### Network integration

Additional global interactions among clusters and modules can be captured by means of the paths and distances between nodes [8]. Shorter paths between brain regions represent stronger potentials for structural integration [24]. The characteristic path length (λ) of the brain network was defined by the average of the shortest path length between individual nodes and other nodes [26]. Like the clustering coefficient, the computed characteristic path length was normalized by the average of the characteristic path lengths of a population of 1,000 randomized networks. Meanwhile, the global network efficiency (E) was computed as the harmonic mean of the inverse values of the shortest path lengths in order to represent the capacity of the network to exchange information [28–30]. While these two integration measures provide complementary information for the network-wide coordination mediating information flow among the direct routes, the global efficiency measure is known to more robustly detect communication distances within networks because it can be computed even when the paths between nodes are disconnected [24].

#### The optimal balance of network segregation and integration

A small-world network represents a network of highly clustered nodes with short node-to-node distances [26]. Network small-worldness (σ) is defined by the ratio of the normalized clustering coefficient and the normalized characteristic path length–*i*.*e*., σ = γ/λ [31]. While a random network is likely to be less clustered with globally short paths (σ<1), a non-random network like the human brain tends to be highly clustered with shorter paths (σ>1). Although we adopted small-worldness as a measure of optimal balance between network segregation and integration, it should be noted that there are on-going debates about whether the current neuroimaging techniques could capture the small-worldness in the brain networks [25, 32].

### Tract-based spatial statistics (TBSS)

Voxel-based statistical analysis of the previous DTI-derived parameters (FA, MD, RD, and AD) was performed using the tract-based spatial statistics (TBSS) of FSL. First, fractional anisotropy (FA) images from each subject were aligned into Montreal Neurological Institute (MNI) standard space with the FMRIB58_FA template using nonlinear transformation, and averaged to produce a mean FA image. The mean FA image was thinned to create a mean FA skeleton, which corresponds to centers of fiber tracts common to the whole group, thresholded at FA>0.2. FA values for each subject were projected onto the mean FA skeleton and analyzed for voxel-wise between-group comparisons. Statistical tests to detect differences in FA between T2DM and control subjects were performed using a nonparametric permutation test with 10,000 Monte Carlo simulations, because the null distribution for the computed diffusion parameter is unknown. Threshold-free cluster enhancement (TFCE) was applied to find significant clusters of voxels (*P*<0.05) and correct multiple comparisons for family-wise error (FWE). MD, AD and RD images were also subjected to the transformations computed from the alignment of FA images, and the same analysis was performed to identify significant clusters for each measure. Anatomical locations of the significant clusters were defined by the maximum statistics at cluster peaks using ICBM-DTI-81 WM atlas and JHU WM tractography atlas.

### Statistics and correlation analysis

The independent two-sample *t*-test was performed to compare differences in global network measures between people with T2DM and their age, sex, education-matched controls. Permutation tests with 10,000 random permutes for each group were performed separately to assess between-group differences on each network measure [22, 23]. Associations between global network measures and plasma glucose (HbA1C and FPG), lipid levels (cholesterol and triglyceride), duration of diabetes, and diabetic microvascular complications were computed using partial correlation coefficients (*r*). A significance level of false discovery rate (FDR) corrected *P*<0.05 was used for all statistics. Age, sex, and the number and strength of connections in the networks were controlled as possible confounding variables to reduce potential effects of inter-subject variability in the connectivity matrix.

## Results

### Changes in the brain network in patients with T2DM

**Fig 2:**
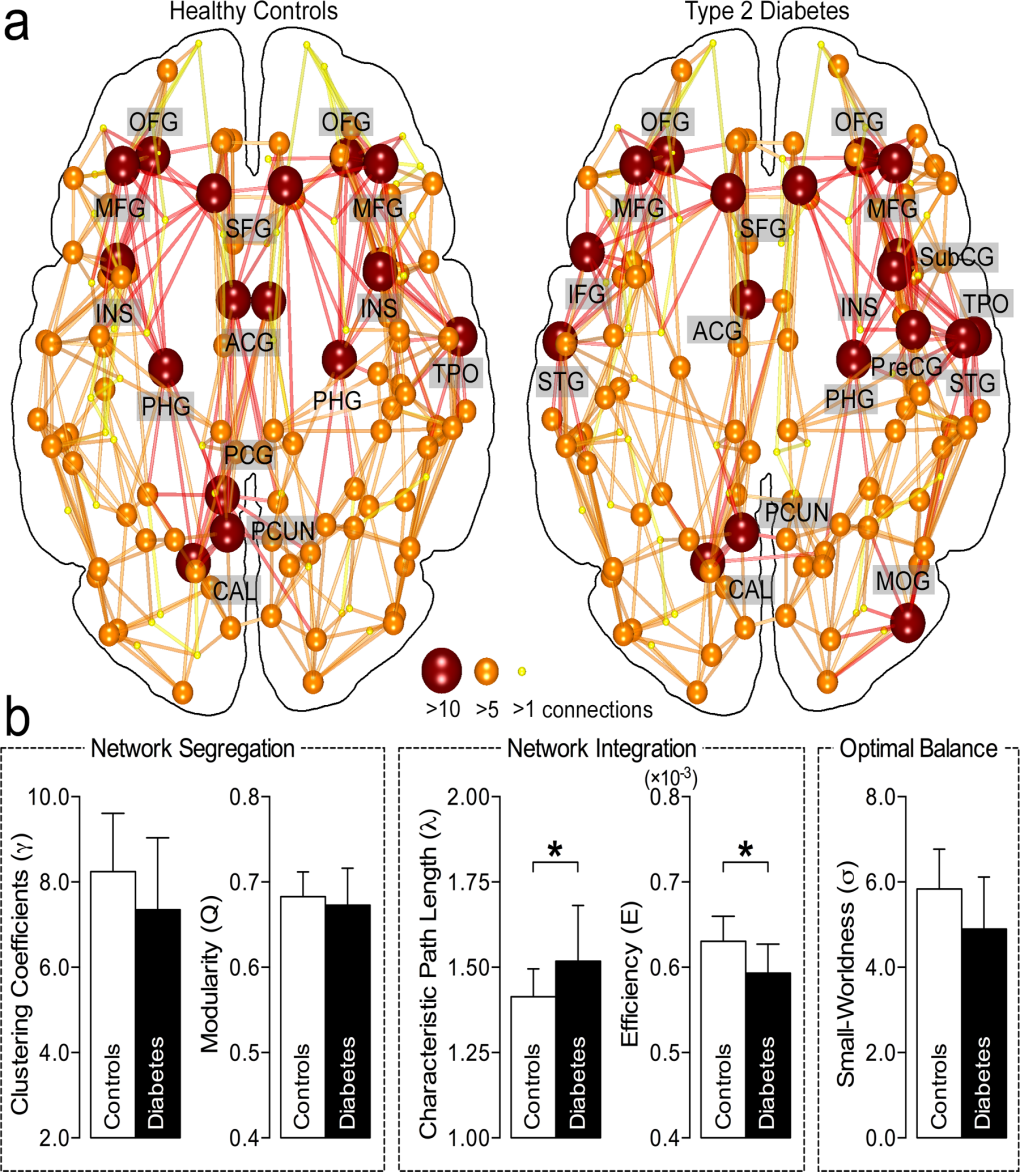
Comparison of brain networks. **a**. The group-averaged structural networks for the controls (left) and type 2 diabetes (right). Nodes (spheres) represent cortical regions based on the brain parcellation, in which the sizes and colors indicate the numbers of connections involving the brain regions. The connections between nodes reflect the reconstructed white matter pathways. The networks vary from person to person, and the lines displayed represent the connections found in at least 75% of the participants. **b**. Structural network properties of the two groups. Middle-aged people with chronic hyperglycemia had a longer path length and lower efficiency than the controls (*FDR corrected *P*<0.05 with 10,000 permutation tests), suggesting impaired network integration in the brains of type 2 diabetes.

In the individuals with diabetes, the measures of network integration showed an abnormality in contrast to those in healthy individuals. However, there was no significant alternation in the network segregation, suggesting no difference with healthy individuals. Fig 2A shows the group-averaged structural networks for the controls (left) and type 2 diabetics (right). Nodes represent cortical regions from the brain parcellation, where the sizes and colors of the nodes indicate the number of connections of each brain region. The connections between nodes reflect reconstructed white matter pathways. In Fig 2B, structural brain networks of the individuals with T2DM and healthy controls had a small-world architecture, *i*.*e*., a higher level of network clustering (γ>1) with shorter paths (λ~1) than a random network, so resulting in higher small-worldness (σ>1). There were no differences (FDR-corrected *P*>0.05) in clustering coefficient (γ_HC_; 8.24±1.37 *vs*. γ_DM_; 7.35±1.68), modularity (Q_HC_; 0.68±0.03 *vs*. Q_DM_; 0.67±0.04), and small-worldness (σ_HC_; 5.84±0.93 *vs*. σ_DM_; 4.90±1.22) (Fig 2B). It shows a trend of decreasing small-worldness of T2DM patients due to the decreased γ and increased λ, but not indicating the severe disruption of small-world network architecture. Also the number of total connections (*P*>0.05) and the average length of connections (*P*>0.05) within the networks did not differ between the two groups (data not shown). These observations suggest that there is no significant alteration in the network segregation in people with poorly controlled diabetes. In contrast, the measures of network integration in the group with diabetes revealed a longer characteristic path length between clusters (λ_HC_; 1.41±0.08 *vs*. λ_DM_; 1.52±0.16, FDR corrected *P*<0.05) and a lower global network efficiency (E_HC_; 0.63±0.03 [×10^−3^] *vs*. E_DM_; 0.59±0.03 [×10^−3^], FDR corrected *P*<0.0005) than in the healthy controls (Fig 2B), suggesting that global network integration is impaired in individuals with diabetes.

### Correlations between parameters of the brain network and glycemic control

**Fig 3:**
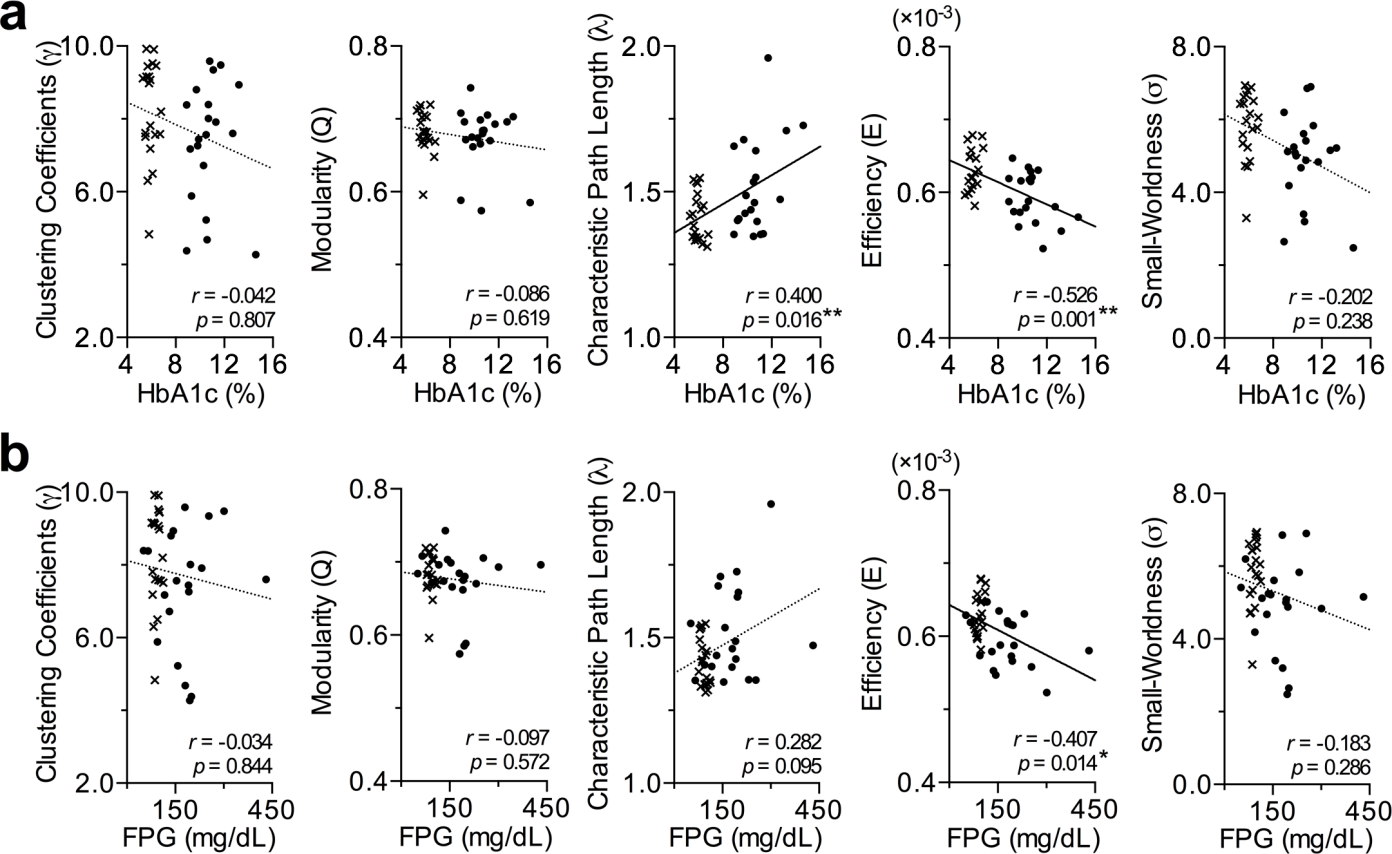
**Correlations between the computed network measures and (a) HbA1c and (b) FPG.** The HbA1c showed significant associations with characteristic path length and network efficiency (**FDR corrected *P*<0.05; solid lines), while FPG had a negative correlation with network efficiency (*uncorrected *P*<0.05; solid line), suggesting that disrupted network integration of the brain structure is associated with increased blood glucose levels. Dots and crosses represent T2DM patients and healthy controls, respectively.

Higher HbA1c levels were associated with a longer network path length (λ; *r* = 0.40, FDR corrected *P*<0.05) and lower network efficiency (E; *r* = −0.53, FDR corrected *P* = 0.001), pointing to disruption of the optimal structural integration in the brains of individuals with T2DM (Fig 3A). Higher FPG levels tend to be correlated with lower network efficiency (*r* = −0.41, *P*<0.05, uncorrected) and longer network path length (*r* = 0.28, *P* = 0.10, uncorrected) although the effect did not reach statistical significance. The parameters of network segregation such as clustering coefficient and modularity were not significantly correlated with HbA1c or FPG. No correlation was found between small-worldness and HbA1c, or between small-worldness and FPG. Age, duration of diabetes, blood pressure, fasting plasma cholesterol and triglyceride concentration or presence of diabetic microvascular complications were not significantly correlated with the parameters of network segregation and integration.

### Correlation between white matter microstructural alteration and glycemic control

**Fig 4:**
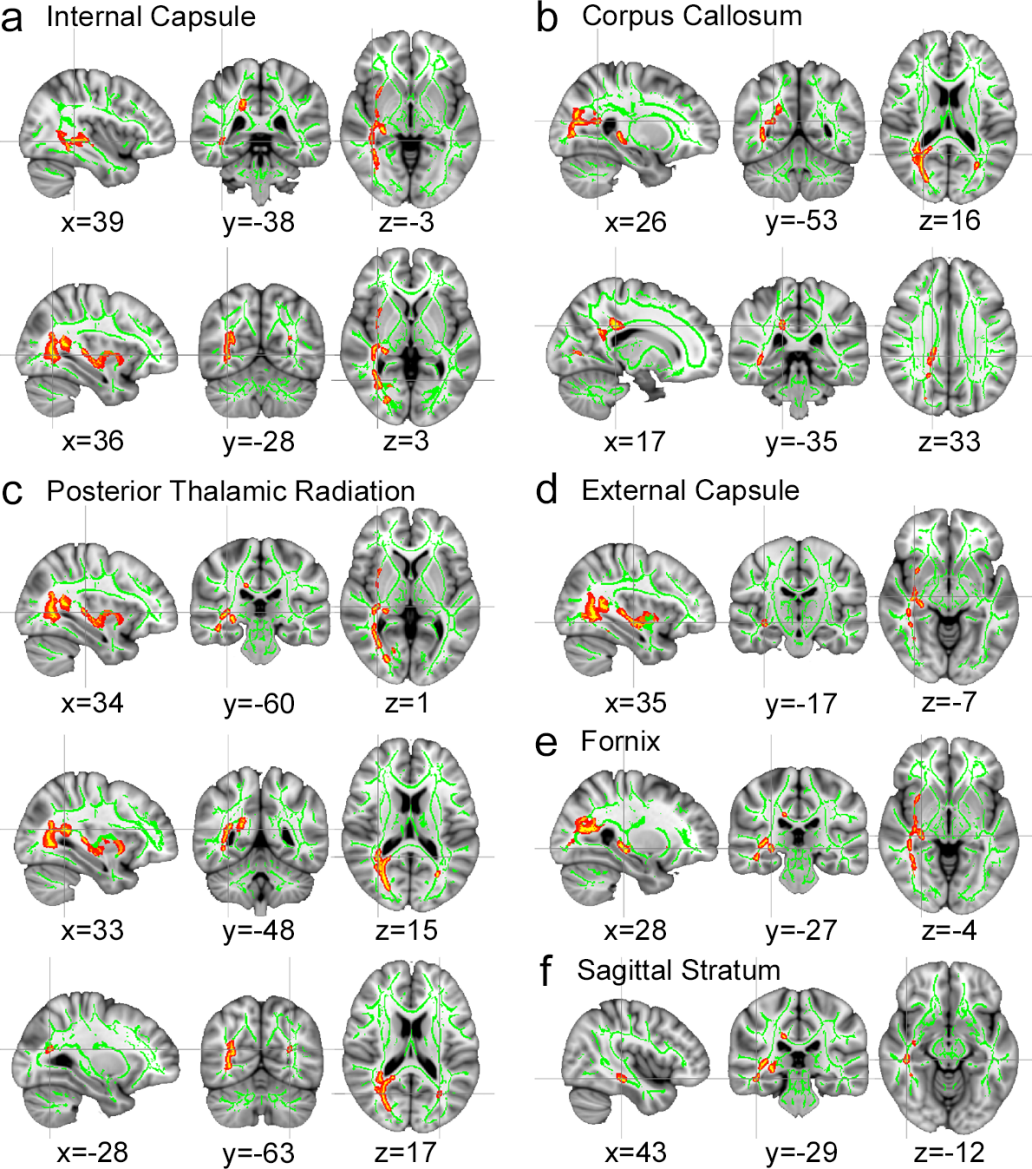
Tract-based spatial statistics (TBSS) results. Results show decreases in fractional anisotropy (FA) in the multiple brain areas of persons with T2DM. Significant TBSS results (red-yellow, *P*<0.05, family-wise error corrected) in sagittal, coronal, and axial views overlaid onto the group averaged FA skeleton (green) and the MNI152 T1 template. The coordinates represent the peak between-group difference in each cluster. At the peaks, negative associations were found between FA value and the blood glucose levels as shown in Fig 3.

To further determine if the altered brain networks in participants with diabetes could be due to microstructural changes in the local regions of white matter, we performed voxel-wise TBSS on DTI images, since FA values are believed to reflect overall health, maturation and organization of white matter [33]. As shown in Fig 4, regional FA values of persons with T2DM were decreased in the distributed white matter regions including projection, commissural, and association fibers (TFCE corrected *P*<0.05). At each cluster peak in Table 2, the FA values were negatively correlated with HbA1c (FDR corrected *P*<0.05) with the specific projection fibers (the posterior thalamic radiation including optic radiation; *P* = −0.45, and the retrolenticular part of the internal capsule; *r* = −0.34), commissural fibers (splenium of the corpus callosum; *r* = −0.47), and association fibers (the fornix; *r* = −0.52, the external capsule; *r* = -0.39, and the sagittal stratum; *r* = −0.53). In addition, negative associations between FPG and regional FA values were found in the retrolenticular part of the internal capsule (*r* = −0.36), splenium of the corpus callosum (*r* = −0.45), fornix (*r* = −0.48), and external capsule (*r* = −0.40). However, there were no significant tract-specific diffusivity differences (MD, AD, and RD) between two groups at TFCE *P*<0.05.

**Table 2:** FA decreases at the cluster peaks from TBSS and correlations with HbA1c and FPG in T2DM patients.

Anatomical locations^¶^		MNI coordinates (mm)			Size (mm^3^)	*t*-value^§^	Correlation (*r*) with	
		*x*	*y*	*z*			HbA1c	FPG
*Projection fibers*								
	PTR including optic radiation (R)	34	-60	1	186	3.374	-0.454*	-0.288
	PTR including optic radiation (R)	33	-48	15	117	3.085	-0.378*	-0.237
	PTR including optic radiation (L)	-28	-63	17	22	3.946	-0.540*	-0.312
	Retrolenticular part of internal capsule (R)	36	-28	3	32	2.799	-0.343*	-0.362*
	Retrolenticular part of internal capsule (R)	39	-38	-3	4	2.803	-0.377*	-0.433*
*Commissural fibers*								
	Splenium of corpus callosum (R)	26	-53	16	358	3.071	-0.470*	-0.454*
	Splenium of corpus callosum (R)	17	-35	33	70	2.636	-0.350*	-0.205
*Association fibers*								
	Fornix (cres) / Stria terminalis (R)	28	-27	-4	89	3.568	-0.523*	-0.480*
	Sagittal stratum including ILF and IFOF (R)	43	-29	-12	28	3.279	-0.526*	-0.340
	External capsule (R)	35	-17	-7	9	2.059	-0.387*	-0.405*

^¶^ Anatomical locations were defined from ICBM-DTI-81 WM atlas and JHU WM tractography atlas.

^§^ The effects are corrected for multiple comparisons by threshold-free cluster enhancement (TFCE) with *P*<0.05.

* Statistical significance was determined at *P*<0.05 (FDR corrected) controlling subject’s age, sex, and the number and strength of connections in the networks.

*Abbreviations*: DTI, diffusion tensor imaging; FPG, fasting plasma glucose; HbA1c, glycated hemoglobin A1c; ICBM, International consortium for brain mapping; IFOF, inferior fronto-occipital fasciculus; ILF, inferior longitudinal fasciculus; JHU, Johns Hopkins University; L, left hemisphere; MNI, Montreal neurological institute; PTR, posterior thalamic radiation; R, right hemisphere; WM, white.

Global FA values of the whole white matter regions (DM; 0.35±0.01, HC; 0.35±0.02) and the extracted white matter tracts from fiber tractography (DM; 0.26±0.01, HC; 0.25±0.02) did not differ between two groups, nor were they correlated with HbA1c and FPG.

## Discussion

We found less integrated brain organization as measured by longer characteristic path length and lower global network efficiency in adults with poorly controlled T2DM in comparison to those in healthy subjects (Fig 2B). The longer path length and the lower global network efficiency were correlated with higher level of HAb1c and FPG in the whole populations (Fig 3). Contrary to the impaired network integration, the parameters of network segregation were not altered in the patients with T2DM.

Segregation and integration are two complementary principles of brain organization. Brain is segregated into highly specialized modules for rapid and automatic information processing as seen in the movement control in the primary motor cortex or early visual input analysis in the occipital cortex [9]. In contrast, integrated brain networks are necessary for consciously effortful cognitive processes such as IQ tests recruiting distributed brain networks [23, 34]. Decreased integration and increased segregation have been observed in the brain networks of the patients with chronic neurological disorders including Alzheimer’s disease and multiple sclerosis, possibly since the pathology preferentially affects the hub nodes linking the modules of the networks [35]. The hub areas with long-distance connections are metabolically expensive with higher wiring cost and thus more vulnerable to oxidative stress than other areas [9]. Microvascular structural impairment in poorly controlled diabetes mellitus [36] could involve hub regions of the brain networks susceptible to this oxidative stress, and would result in disruption in the integration of the brain networks.

Cognitive performance is thought to be mediated by multiple interacting brain circuits and their connections [37]. Network path length and efficiency represent how efficiently a node (*i*.*e*., a parcellated brain region) is connected to the remaining nodes [24] and thus may reflect the efficiency of information transfer in the network [28]. Widespread deterioration of the brain network has previously been shown to reflect an age-related reduction in information-processing speed [38] and functional network reorganization [39]. Psychomotor slowing was significantly associated with poor glycemic control [40]. Although we did not assess cognitive performance, the slowing of information processing was a prominent cognitive feature in patients with type 2 diabetes and similar demographic and metabolic characteristics as our cohort—middle-aged adults (mean age 50.8 ± 7.7 years) with poorly controlled T2DM (HbA1c 10.2 ± 2.4%) [41]. Using DTI and graph theory, Reijmer and colleagues have recently shown that mental slowing is related to decreased network efficiency and increased shortest path length [11]. Therefore, altered WM network topology that we observed in the study provides an explanation for the slowing of information processing in subjects with diabetes.

On the other hand, we found a significant reduction in the FA values of the widespread WM tracts in individuals with diabetes. The FA values in the multiple WM tracts including the optic radiation, the internal and external capsules, the splenium of the corpus callosum, the fornix, and the sagittal stratum were inversely correlated with HbA1c. In line with our findings, a significant reduction in FA values has been reported in the splenium of the corpus callosum, the internal capsule, the external capsule, and optic radiations in type 1 and type 2 diabetic patients [2, 3] and lower FA values were reported to be associated with longer duration of diabetes in type 1 diabetes [42]. Along with the disruption in the integration of the brain networks, the widespread disruption of WM fiber integrity found in the current study could be a “central neuropathy induced by chronic hyperglycemia” [40].

Some limitations of this study should be taken into account. The sample size for each group was relatively small (*n* = 20) due to difficulty in recruiting subjects with poor diabetes control. Also the group with diabetes included more smokers than the control group although other clinical variables [*e*.*g*., age, gender, education, cholesterol, and body mass index (BMI)] did not differ between the diabetic and control groups. Furthermore, we did not examine cognitive/behavioral characteristics in our participants of the study.

In summary, using DTI and graph theory, we found that poorly controlled hyperglycemia disrupts the topology of the brain network in T2DM. The measures of network integration showed decreased global efficiency and increased path length in patients with T2DM compared to those in healthy subjects and the measures had a correlation with the level of hyperglycemia. We suggest that disrupted network integration with widespread disruption of WM fiber integrity represent a manifestation of central neuropathy in diabetes, possibly contributing to mental slowing and cognitive impairments in individuals with diabetes.
